# Transmitted HIV-1 drug resistance and subtype distribution among treatment-naïve individuals at a tertiary referral centre in the Aegean Region of Türkiye: an eight-year cohort study

**DOI:** 10.1007/s15010-026-02810-1

**Published:** 2026-05-11

**Authors:** İlker Ödemiş, İlkay Akbulut, Esra Duru Öz, Ahmet Can Harput, Büşra Yaren Özcan, Tülin Demir, Sabri Atalay

**Affiliations:** 1 Department of Infectious Diseases and Clinical Microbiology, Tepecik Training and Research Hospital, University of Health Sciences, Izmir, Türkiye; 2 Department of Medical Microbiology, School Of Medicine, Ahi Evran University, Kırşehir, Türkiye

**Keywords:** HIV-1 transmitted drug resistance, Antiretroviral resistance mutations, NNRTI resistance, Integrase strand transfer inhibitor resistance, HIV-1 subtype distribution, Türkiye

## Abstract

**Purpose:**

Transmitted drug resistance (TDR) threatens the effectiveness of first-line antiretroviral therapy, yet contemporary regional data from the Aegean region of Türkiye remain limited. This study evaluated TDR prevalence, mutational profiles, and HIV-1 subtype distribution among treatment-naïve individuals.

**Methods:**

We conducted a single-center retrospective cohort study at a tertiary referral center in İzmir, Türkiye, including adults with confirmed HIV-1 infection who were antiretroviral-naïve at baseline between January 2017 and December 2024. Genotypic resistance testing was performed before treatment initiation using Sanger sequencing. Resistance mutations were interpreted with Stanford HIVdb v10.1, and TDR was defined as a penalty score of ≥ 10 for at least one drug.

**Results:**

A total of 163 individuals were included; the mean age was 38.16 ± 12.48 years, 91.4% were male, and subtype B was the most common clade (50.3%). Overall, 29 individuals (17.8%) had at least one resistance-associated mutation. NNRTI resistance predominated (15.3%), followed by NRTI (1.8%) and PI (0.6%); no INSTI mutations were detected. HIV-1 subtype F remained independently associated with antiretroviral resistance in multivariable logistic regression (OR 31.4, 95% CI 6.10–335.1; *p* < 0.001). E138A was the most common NNRTI substitution (7.4%). S68G was the most frequent accessory mutation, identified in 41.7% of patients.

**Conclusion:**

Transmitted drug resistance remains clinically relevant in treatment-naïve individuals in the Aegean region of Türkiye and is driven predominantly by NNRTI-associated mutations. The absence of baseline INSTI resistance supports the preserved activity of contemporary INSTI-based first-line regimens, while the observed association between subtype F and resistance warrants further molecular surveillance.

## Introduction

Human immunodeficiency virus (HIV) infection remains a major global public health concern despite the remarkable advances achieved with antiretroviral therapy (ART). The most recent UNAIDS estimates place the number of people living with HIV at approximately 40.8 million worldwide in 2024, with nearly 1.3 million new infections occurring annually; significant progress has been made toward the 95–95–95 targets, and these gains have translated into meaningful reductions in HIV-related morbidity and mortality globally [1]. While this mass expansion of ART coverage creates a significant selective pressure for the evolution of resistant variants and their transmission to ART-naïve populations, concurrent challenges in accessing antiretroviral drugs in recent years—particularly in low- and middle-income countries—have led to declines in treatment adherence, further accelerating the emergence of antiretroviral drug resistance [2]. Of greater clinical concern, the transmission of these resistant strains to treatment-naïve individuals threatens to compromise the efficacy of modern first-line treatment backbones from the very onset of therapy. Consequently, ongoing molecular surveillance of transmitted drug resistance (TDR) is a critical imperative to safeguard the long-term sustainability of the global 95–95–95 targets.

Antiretroviral drug resistance can either emerge during treatment or be transmitted to previously untreated individuals, a phenomenon termed TDR [3]. The prevalence of TDR varies considerably by region and income level; rates are generally reported to be below 10% in many resource-limited settings, whereas they frequently exceed 10–15% in high-income countries, with Southern and Eastern European countries typically falling somewhere in between [4–6]. In Türkiye, the HIV epidemic is expanding rapidly, with 54,472 cumulative HIV-positive individuals reported as of November 2025 [7]. Recent data from a major tertiary center in Central Anatolia indicated a TDR prevalence of 14.42% among newly diagnosed patients, with non-nucleoside reverse transcriptase inhibitor (NNRTI) resistance constituting the predominant class at 7.69% [8]. In contrast, the most recent regional data from the Aegean region, which covered the period up to 2019, reported a much lower TDR rate of 4.1% [5]. This substantial discrepancy between different geographic cohorts, alongside the lack of post-2019 surveillance, highlights a critical gap in the literature. To our knowledge, no contemporary data exist evaluating how the shifting dynamics of the epidemic and the introduction of modern ART regimens have shaped the TDR landscape in the Aegean region over the last five years.

This study aimed to characterize the prevalence and mutational profiles of TDR across major antiretroviral classes, including integrase inhibitors, and to evaluate HIV-1 subtype distribution among treatment-naïve individuals at a tertiary center in the Aegean region of Türkiye between 2017 and 2024.

## Methods

### Study design and population

This was a single-center retrospective observational study conducted in the Department of Infectious Diseases and Clinical Microbiology at İzmir Tepecik Training and Research Hospital, a tertiary referral center serving the Aegean region of Türkiye. The study period spanned from 1 January 2017 to 31 December 2024. The study population comprised adults aged 18 years or older with a confirmed HIV-1 diagnosis who were ART-naïve at the time of their initial presentation. ART-naïve status was verified at the time of diagnosis based on clinical history, with particular attention to prior exposure to antiretrovirals in any context, including pre-exposure prophylaxis (PrEP) and prevention of mother-to-child transmission (PMTCT) regimens; individuals with any prior antiretroviral exposure were excluded. Genotypic resistance testing was performed once at baseline, prior to ART initiation, as part of routine clinical care. Patients younger than 18 years of age, those with incomplete clinical records, and those for whom genotypic sequencing was unsuccessful were excluded from the analysis. Antiretroviral drugs were grouped into four major classes: nucleoside/nucleotide reverse transcriptase inhibitors (NRTIs), NNRTI, protease inhibitors (PIs), and integrase strand transfer inhibitors (INSTIs). Resistance-associated mutations were analyzed separately for each class.

### Data collection

The following variables were recorded: age, sex, nationality, comorbidities, transmission route, history of smoking, alcohol or substance use, and resistance test results in patients. CD4+ T lymphocyte counts and HIV-1 RNA levels measured on the day of resistance testing were recorded.

### Genotypic resistance testing

Drug resistance mutations were interpreted using the Stanford University HIV Drug Resistance Database (HIVdb, version 10.1). TDR was defined as the presence of any mutation conferring a Stanford HIVdb penalty score of ≥ 10 for at least one drug within each class.

Mutations with little or no demonstrated effect on drug susceptibility were classified as accessory mutations; this term denotes mutations that reduce susceptibility only in combination with a major resistance mutation, or that increase the replication fitness of viruses harbouring major drug resistance mutations [9]. Sequences exhibiting mixed base calls on chromatographic analysis were reprocessed from RNA extraction through repeat sequencing; only unambiguous, high-quality chromatograms were included in the final analysis. Accessory mutations were reported descriptively but were not included in the calculation of TDR prevalence.

### Nucleic acid extraction and amplification

Viral RNA was isolated from plasma samples using the automated MagNA Pure extraction system (Roche Molecular Diagnostics, USA). Following extraction, RNA samples were stored at −80 °C until further processing. Reverse transcription and primary amplification were performed using the SuPrimeScript one-step RT-PCR kit (Genetbio, South Korea). Each reaction was prepared in a final volume of 25 µL containing 0.8 µM of each primer. The thermal cycling protocol consisted of reverse transcription at 50 °C for 40 min, followed by an initial denaturation step at 95 °C for 15 min. Subsequently, 35 amplification cycles were carried out with denaturation at 94 °C for 30 s and annealing for 30 s at 53 °C for the protease/reverse transcriptase (PR/RT) regions and 50 °C for the integrase (IN) region. Extension was performed at 72 °C for 150 s for the PR/RT region and 90 s for the IN region. The amplification process concluded with a final extension step at 72 °C for 10 min. Nested PCR was subsequently performed to further amplify the PR, RT, and IN regions using HS Prime Taq Premix (Genetbio, South Korea). Each nested PCR reaction was prepared in a 25 µL mixture containing 0.8 µM of each primer. Cycling conditions included an initial denaturation at 95 °C for 15 min, followed by 35 cycles of denaturation at 94 °C for 30 s and annealing for 30 s at 53 °C for the PR/RT regions and 55 °C for the IN region. Extension was performed at 72 °C for 150 s for the PR/RT region and 90 s for the IN region, followed by a final extension step at 72 °C for 10 min. Amplified products were visualized by electrophoresis on 1.5% agarose gels.

### Sequencing, sequence analysis and subtype determination

Successful amplicons were subjected to Sanger sequencing using the BigDye™ Terminator v3.1 Cycle Sequencing Kit (Thermo Fisher Scientific, USA) and analyzed on an Applied Biosystems™ 3500xL Genetic Analyzer (Thermo Fisher Scientific, USA).

Sequence editing, assembly, and quality assessment were performed using the Unipro UGENE software package. HIV-1 subtype determination was performed using the REGA HIV-1 Subtyping Tool (version 3.0). Subtype assignment was based on partial pol gene sequences, including protease (codons 1–99), reverse transcriptase (codons 1–346), and integrase (codons 1–288) regions. In selected cases, subtype determination was additionally verified using the Stanford HIVdb Subtyping Tool, with concordant results. Subtype assignments were interpreted comparatively across the three genomic regions to identify potential discordance indicative of recombinant structures. Drug resistance mutations were determined using the Stanford University HIV Drug Resistance Database (HIVdb, version 10.1). Primer sequences for the protease/reverse transcriptase and integrase regions were used as described by the WHO and ANRS AC43 HIV Resistance Study Group, respectively [10, 11].

**Table 1:** Primers used in the study

Region	Primer name	Primer sequence (5’-3’)
RT-PCR Primers		
Protease*	PR1_F	TGAARGAATTGYACTGARAGRCAGGCTAAT
Protease*	PR2_R	AYCTIATYCCTGGTGTYTCATTRTT
Reverse transcriptase*	RT1_F	TTTYAGRRGARCTYAATAARAGAACTCA
Reverse transcriptase*	RT2_R	CCTCITTYTGCATAYTTYCCGTGT
Integrase†	INPS1	TAGTAGCCAGCTGTGATAAATGTC
Integrase†	INPR8	TCCCATGTTCTAAATCCTCATCCTG
Nested-PCR primers		
Protease*	PR3_F	YTCAGRCAGRCCRGARCCAACAGC
Protease*	PR4_R	CTGGTGTYTCATTRTTKTRTACTAGGT
Reverse transcriptase*	RT3_F	TTYTGGGARGTYCARYTAGGRATACC
Reverse transcriptase*	RT4_R	GGYTCTTGTAAAATTTGRTATGTCC
Integrase†	INPS3	GAAGCCATGCATGGACAAG
Integrase†	INPR9	ATCCTCATCCTGTCTACTTGCC

Values marked with * and † were obtained from References 10 and 11, respectively.

### Statistical analysis

The primary outcome was the presence of at least one resistance-associated mutation, defined as a Stanford HIVdb penalty score ≥ 10 for any drug within any antiretroviral class. Categorical variables were expressed as numbers and percentages, whereas continuous variables were presented as mean ± standard deviation (SD) or median with interquartile range (IQR) depending on data distribution. Normality of continuous variables was assessed using the Shapiro–Wilk test. For categorical variables, Pearson’s chi-square test or Fisher’s exact test was used as appropriate. Continuous variables with normal distribution were analyzed using the Student’s t-test, whereas the Mann–Whitney U test was used for variables that did not meet the normality assumption. Subtype F, which was identified as significantly associated with antiretroviral drug resistance in univariable analysis, was further evaluated in a multivariable Firth penalized logistic regression model. Age and sex were included as a priori covariates based on their established clinical relevance as potential confounders in HIV drug resistance analyses, irrespective of their univariable significance. Given the small number of subtype F infections and the risk of complete separation inherent to standard binary logistic regression, Firth penalized logistic regression was applied to obtain stable and unbiased estimates of effect. Results were reported as odds ratios (ORs) with 95% confidence intervals (95% CIs). Firth regression was performed using the logistf package (version 1.26) in R (version 4.5.0); all remaining statistical analyses were performed using SPSS version 27.0 (IBM Corp., Armonk, NY, USA). A two-sided p-value of < 0.05 was considered statistically significant.

## Results

### Study population

A total of 163 patients were enrolled in the study; the mean age was 38.16 ± 12.48 years, and the majority were male (*n* = 149, 91.4%). Most participants were Turkish citizens (*n* = 157, 96.3%). Men who have sex with men (MSM) represented the most common transmission category (*n* = 72, 44.1%), followed by heterosexual contact (*n* = 70, 43%), and unknown route (*n* = 21, 12.8%). Baseline demographic, behavioral characteristics, and comorbidities are summarized in Table 2.

**Table 2:** Demographic characteristics, transmission routes, and comorbidities according to resistance status

	Total *n* = 163 (%)	Resistance-Associated Mutations		*p* value
		Negative *n* = 134 (82.2%)	Positive *n* = 29 (17.8%)	
Age (year)*	38.16 ± 12.48	38.63 ± 12.20	35.97 ± 13.68	0.298
Male	149 (91.4%)	121 (90.3%)	28 (96.6%)	0.468
Single/Divorced Married	111 (68.1%) 52 (31.9%)	91 (67.9%) 43 (32.1%)	20 (69%) 9 (31%)	0.912
Turkish Foreign national	157 (96.3%) 6 (3.7%)	128 (95.5%) 6 (4.5%)	29 (100%) 0	0.592
Alcohol use	80 (49.1%)	67 (50%)	13 (44.8%)	0.613
Tobacco use	103 (63.2%)	84 (62.7%)	19 (65.5%)	0.774
Substance use	25 (15.3%)	20 (14.9%)	5 (17.2%)	0.778
Transmission Route				
MSM	72 (44.1%)	58 (43.3%)	14 (48.3%)	0.624
Heterosexual	70 (43%)	59 (44%)	11 (37.9%)	0.547
Unknown	21 (12.8%)	17 (12.7%)	4 (13.8%)	0.759
Comorbidities				
Diabetes mellitus	7 (4.3%)	5 (3.7%)	2 (6.9%)	0.609
Hypertension	11 (6.7%)	9 (6.7%)	2 (6.9%)	1.0
CKD	7 (4.3%)	5 (3.7%)	2 (6.9%)	0.609
CVD	9 (5.5%)	7 (5.2%)	2 (6.9%)	0.662
Neurological disease	3 (1.8%)	2 (1.5%)	1 (3.4%)	0.447
Psychiatric disease	27 (16.6%)	19 (14.2%)	8 (27.6%)	0.098
Hyperlipidemia	20 (12.3%)	15 (11.2%)	5 (17.2%)	0.359
HIV-RNA (copies/mL)**	135.000 (35,100–386,000)	111.500 (28,900–351,250)	177,000 (49,650–488,000)	0.159
CD4 + T-lymphocyte count/mm^3^*	420.98 ± 235.66	412.93 ± 234.88	458.14 ± 239.88	0.351
CD4 + T-lymphocyte percentage/%*	21.87 ± 10.38	21.86 ± 10.28	21.89 ± 10.99	0.988

MSM: Men who have sex with men, CKD: Chronic kidney disease, CVD: Cardiovascular disease, * mean and ± SD, ** median and IQR (25–75)

The mean CD4 + T-cell count was 420.98 ± 235.66 cells/mm³, with a mean CD4% of 21.8 ± 10.38%. The median HIV RNA level was 135,000 copies/mL (IQR: 35,100–386,000). Subtype B was the most prevalent clade, accounting for 82 infections (50.3%). The remaining cases comprised a heterogeneous mix of non-B subtypes and recombinant forms, with A6 (8.0%), G (5.5%), F (4.9%), and C (4.3%) representing the most common pure non-B subtypes. Mixed or recombinant infections accounted for 44 cases (27.0%). The complete subtype distribution is detailed in Table 3.

**Table 3:** Distribution of HIV subtypes

HIV Subtype	Total *n* = 163 (%)	Resistance-Associated Mutations		*p* value
		Negative *n* = 134 (82.2%)	Positive *n* = 29 (17.8%)	
B*	82 (50.3%)	69 (51.5%)	13 (44.8%)	0.515
A6*	13 (8.0%)	12 (9.0%)	1 (3.4%)	0.467
G*	9 (5.5%)	7 (5.2%)	2 (6.9%)	0.662
F*	8 (4.9%)	1 (0.7%)	7 (24.1%)	**< 0.001**
C*	7 (4.3%)	6 (4.5%)	1 (3.4%)	1.000
Total Recombinant/Mixed Subtypes	44 (27.0%)	39 (29.1%)	5 (17.2%)	0.192
B + F	17 (10.4%)	15 (11.2%)	2 (6.9%)	0.740
B+CRF02_AG	7 (4.3%)	7 (5.2%)	0	-
B + C	5 (3.1%)	5 (3.7%)	0	-
CRF02_AG	4 (2.5%)	4 (3.0%)	0	-
G+CRF02_AG	3 (1.8%)	3 (2.2%)	0	-
G + J	3 (1.8%)	2 (1.5%)	1 (3.4%)	-
A6/A1	1 (0.6%)	1 (0.7%)	0	-
B/CRF56_cpx	1 (0.6%)	1 (0.7%)	0	-
C/CRF57_BC	1 (0.6%)	1 (0.7%)	0	-
B+CRF02_AG/CRF56_cpx	1 (0.6%)	0	1 (3.4%)	-
G + J/CRF13_cpx	1 (0.6%)	0	1 (3.4%)	-

*Data on patients in whom a single subtype was detected by PCR, data with a “-” sign in the p-value column could not be calculated due to insufficient number of variables

Overall, 29 individuals (17.8%) had at least one drug resistance mutation. Resistance-associated mutations were detected in 25 patients (15.3%) for NNRTIs, three patients (1.8%) for NRTIs, and one patient (0.6%) for PIs.

In the subgroup analysis of treatment-naïve patients, no statistically significant association was observed between antiretroviral resistance and age, sex, marital status, nationality, comorbid conditions, smoking, alcohol or substance use, CD4 count, CD4%, or HIV RNA level (Table 2). In univariable analysis, HIV-1 subtype F was significantly associated with the presence of drug resistance mutations (OR: 41.7, 95% CI: 4.9–333, *p* < 0.001). In multivariable Firth penalized logistic regression adjusting for age and sex, HIV-1 subtype F remained independently associated with antiretroviral drug resistance (OR: 31.4, 95% CI: 6.10–335.1, *p* < 0.001). Among the eight subtype F infections, seven (87.5%) harboured resistance-associated mutations; in all seven cases, E138G was the sole NNRTI-associated mutation detected, with no NRTI, PI, or INSTI resistance mutations identified.

**Fig. 1:**
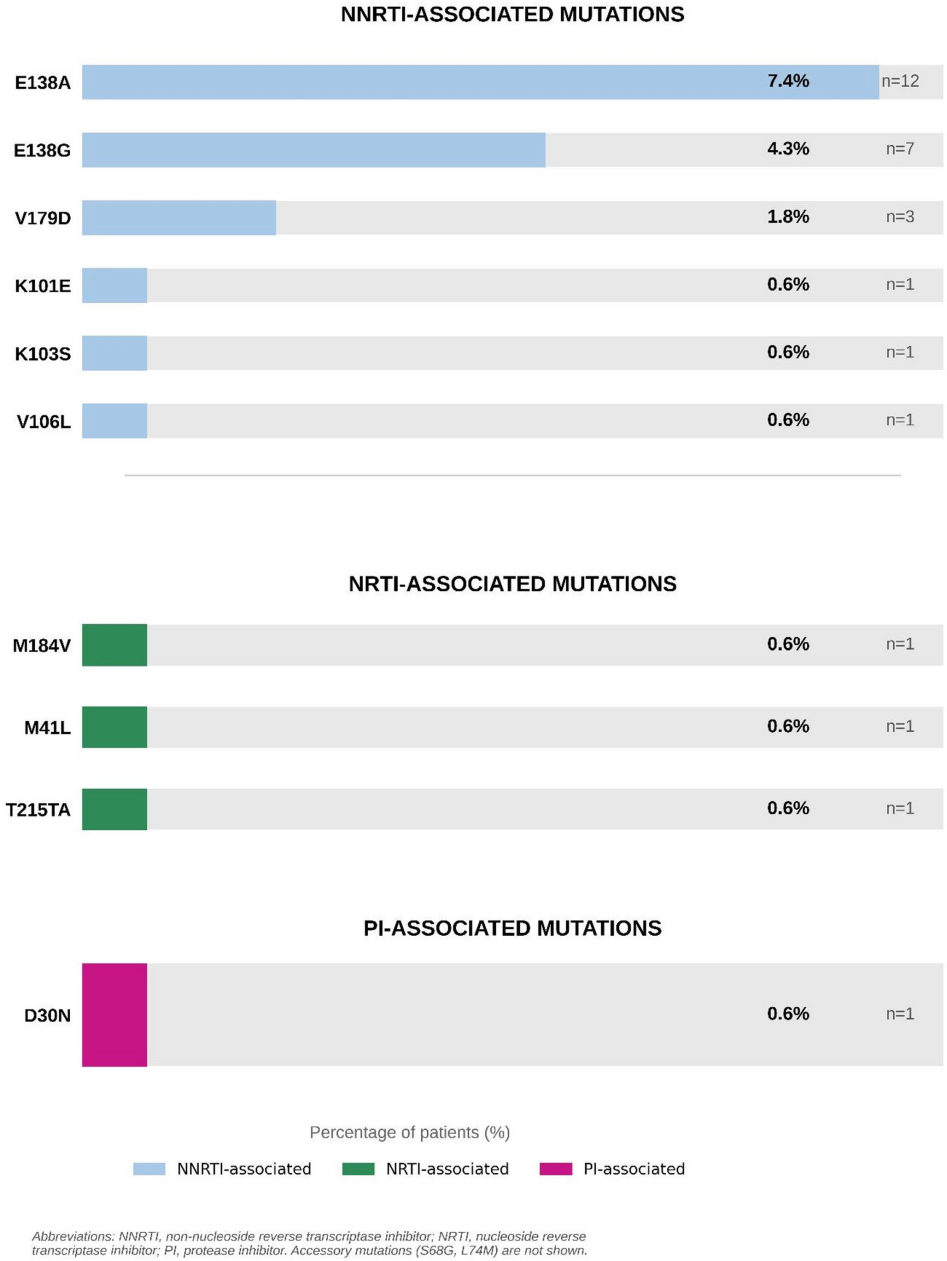
The distribution of resistance mutations

Among all detected mutations, the most frequently observed was the accessory mutation S68G, identified in 68 patients (41.7%). Of the 68 patients with S68G, 49 (72.1%) had HIV subtype B. Regarding NRTI-associated mutations, M184V, M41L, and T215T/A were each detected in one patient (0.6%). Among the mutations associated with NNRTI, E138A was detected in 12 patients (7.4%), E138G in seven patients (4.3%), and V179D in three patients (1.8%). For protease inhibitors, the D30N mutation was detected in one patient (0.6%). No resistance mutations associated with INSTIs were identified. The distribution of resistance mutations is summarized in Fig. 1.

## Discussion

In this cohort from the Aegean region, the overall prevalence of TDR was 17.8%, driven predominantly by NNRTI-associated mutations. Crucially, no major resistance mutations to INSTIs were detected. Our observed TDR rate was higher than that reported in earlier studies from the same region and in some recent Turkish cohorts [5, 8]. This difference may reflect variations in study period, population structure, circulating subtype distribution, and the continued transmission of NNRTI-resistant strains selected during earlier treatment eras.

Consistent with recent Turkish cohorts and international data including the Mediterranean MeditRes cohort, we observed no significant association between TDR and demographic, clinical, immunological, or baseline virological parameters [5, 8, 12]. While MSM represented the predominant transmission route in our cohort, a group frequently identified as a key driver of drug-resistant HIV transmission in Western Europe, MSM status was not independently associated with TDR in our analysis [13, 14]. This finding is consistent with other Turkish cohorts and may reflect genuine regional differences in transmission patterns compared with those reported in Western European cohorts; however, the limited sample size precludes definitive conclusions.

Subtype B accounted for just over half of the infections in our cohort, yet the remaining cases comprised a notably diverse array of non-B subtypes and recombinant forms. This heterogeneity closely mirrors recent molecular epidemiology studies from Türkiye, which report subtype B frequencies of approximately 50% to 55% alongside a growing proportion of non-B variants [5, 8, 15]. The expanding viral diversity in the Aegean region aligns with broader shifts documented across European cohorts, where increasing non-B prevalence is strongly driven by migration and the dynamic integration of new transmission networks [16]. Regarding the relationship between viral clade and drug resistance, previous national cohorts have not identified a statistically significant association between specific subtypes and TDR [5, 8]. In contrast, our multivariable Firth penalized logistic regression indicated an independent association between subtype F and antiretroviral resistance (OR: 31.4, 95% CI: 6.10–335.1, *p* < 0.001). In all seven resistance-positive subtype F patients, E138G was the sole NNRTI-associated resistance mutation detected; no NRTI, PI, or INSTI resistance mutations were identified. While certain non-B subtypes are known to harbor naturally occurring polymorphisms that can modulate the genetic barrier to resistance, the observation that E138G was detected in 87.5% of subtype F isolates—while being absent in other subtypes—suggests that this substitution may, at least in part, reflect a clade-specific genetic pattern rather than true transmitted drug resistance. Although subtype F does not broadly exhibit polymorphism at most mutation-associated resistance positions, the E138 codon represents a recognized exception, as E138 substitutions have been documented as natural polymorphisms across several non-B subtypes [15, 17, 18]. However, the small number of subtype F infections (*n* = 8), the relatively wide confidence interval, and the imprecision of the estimated effect size indicate that the observed association should be regarded as hypothesis-generating rather than definitive.

In our cohort, baseline resistance was predominantly associated with the NNRTI class, primarily driven by substitutions at codon E138, which aligns with findings from recent large-scale Turkish cohorts [15, 19]. K103N, the most prevalent transmitted NNRTI mutation globally, was not detected in our cohort. This observation may reflect the transition in Türkiye from NNRTI-based first-line regimens, predominantly those containing efavirenz, to dolutegravir-based regimens around October 2018, with a consequent reduction in population-level selective pressure for classical NNRTI resistance mutations. The sharp increase in HIV incidence observed in Türkiye following the COVID-19 pandemic implies that a substantial proportion of newly diagnosed individuals in the latter years of our study were likely infected during a period in which NNRTI-based regimens were no longer the standard of care, further diminishing the likelihood of K103N transmission [7]. The replicative fitness cost associated with K103N may also contribute to its decline in the absence of sustained drug pressure. Consistent with this trend, Sertoz et al. reported a K103N prevalence of only 0.9% in an earlier Aegean cohort [5], and Özdemir et al. similarly observed a low prevalence of 1.92% in Central Anatolia [8], supporting a broader pattern of declining K103N transmission across Turkish cohorts. As discussed above, E138 variants frequently occur as natural background substitutions in non-B subtypes [15], and the absence of K103N despite the high overall NNRTI-associated mutation rate supports this interpretation. Although isolated E138 substitutions typically confer only potential low-level resistance to rilpivirine, their accumulation alongside other NNRTI-associated mutations can significantly compromise drug susceptibility [20]. Crucially, baseline rilpivirine resistance—including E138 variants—has emerged as a key predictor of virologic failure in patients transitioning to long-acting cabotegravir-rilpivirine therapy, as demonstrated in the ATLAS and ATLAS-2M trials [21, 22]. The relatively frequent detection of E138-associated mutations in our cohort may narrow the pool of patients eligible for cabotegravir–rilpivirine long-acting therapy and supports careful baseline resistance screening when rilpivirine-based strategies are being considered—regardless of whether these substitutions represent true transmitted resistance or subtype-specific background substitutions.

In our cohort, no transmitted INSTI resistance mutations were identified, consistent with recent Turkish molecular epidemiology studies in which major INSTI mutations among treatment-naïve individuals were either absent or detected only at very low frequencies [5, 8, 15]. Comparable low baseline rates have also been observed across the broader Mediterranean region; for example, the multicenter MeditRes study reported an INSTI surveillance drug resistance mutation prevalence of only 0.3% [12]. Although baseline INSTI susceptibility appears to be preserved in our setting, increasing global reports of acquired dolutegravir resistance among treatment-experienced individuals underscore the importance of continued molecular surveillance [23]. Taken together, these findings support the preserved activity of contemporary INSTI-based regimens in treatment-naïve individuals.

Transmitted PI resistance was exceptionally rare in our cohort, restricted to a single individual harboring the major D30N mutation. This aligns with recent national surveillance data demonstrating negligible baseline PI resistance across Türkiye [5, 8]. This consistently low prevalence reflects both the inherently high genetic barrier of protease inhibitors and their declining clinical use in the INSTI era, which has substantially diminished the population-level selective pressure required for the emergence and onward transmission of such variants. Because D30N exclusively confers resistance to nelfinavir, its clinical relevance is currently obsolete. Nevertheless, its detection illustrates how historical drug-resistant lineages can persist within local transmission networks in the absence of ongoing selective drug pressure.

Transmitted NRTI resistance was uncommon in our cohort, presenting at a lower prevalence than recent national estimates from Central Anatolia but remaining consistent with the low, persistent circulation previously documented in the Aegean region [5, 8]. Notably, the identification of M41L and T215 revertants (T215T/A) in consecutive Aegean cohorts suggests the persistent circulation of historical thymidine analogue-associated mutations within regional transmission networks [5]. As T215 revertants emerge from T215Y/F to restore viral fitness in the absence of drug pressure, their presence serves as a critical biomarker of primary resistance transmission rather than a direct determinant of clinical failure [24]. The detection of the M184V major mutation is particularly noteworthy. Given its severe replicative fitness cost and rapid reversion kinetics in the absence of drug pressure, baseline M184V strongly implies a recent transmission event from a source partner experiencing virological failure on a lamivudine/emtricitabine-containing regimen. Altogether, these mutational profiles underscore that while clinically relevant variants continue to circulate locally, the overall low prevalence of NRTI resistance reaffirms the sustained efficacy of contemporary NRTI backbones, supporting their continued reliable use in first-line antiretroviral regimens.

Although S68G does not independently confer antiretroviral resistance, it has been described as a compensatory accessory substitution that partially restores the replication fitness defect associated with K65R [25]. S68G was the most frequently detected accessory mutation in our cohort, identified in 41.7% of individuals — a prevalence substantially exceeding the 1.6–7.2% reported in global treatment-naïve populations [26–28]. Among patients carrying S68G in our cohort, 72.1% were infected with HIV-1 subtype B, and Özdemir et al. similarly reported that 77.59% of individuals with S68G belonged to subtype B [8]. The consistency of these findings suggests that the high frequency of S68G in Türkiye may be influenced, at least in part, by the subtype composition and local genetic background of circulating strains. While the clinical significance of isolated S68G remains limited, its high prevalence warrants continued surveillance, as co-emergence with K65R could accelerate resistance compensation in this population.

This study has several limitations that merit consideration. Epidemiologically, the single-center, retrospective design and relatively modest sample size may restrict the broader generalizability of our findings and introduce potential information bias. Specifically, the small number of non-B infections limits the precision of clade-specific estimates; the relatively wide confidence interval observed for subtype F, even after applying Firth penalized regression to address separation bias, directly reflects this sample size constraint and limits the precision of the effect estimate. Furthermore, the absence of phylogenetic cluster analysis precludes us from confirming whether the disproportionate concentration of resistance mutations among subtype F infections originated from a common transmission network rather than a true biological vulnerability. As E138G was the sole resistance mutation detected in all resistance-positive subtype F isolates, and E138 substitutions are recognized as natural polymorphisms in several non-B subtypes, the observed association between subtype F and TDR may partly be attributable to a clade-specific polymorphic pattern rather than genuine transmitted drug resistance. Methodologically, our reliance on a single automated tool (REGA) for subtype determination, without maximum-likelihood phylogenetic reconstruction, might oversimplify the detection of complex recombinant forms. Additionally, genotypic resistance testing was performed using population-based Sanger sequencing, which intrinsically fails to detect minority resistance variants present below the 20% detection threshold.

## Conclusion

In conclusion, our findings indicate that transmitted antiretroviral drug resistance remains a clinically relevant issue in the Aegean region of Türkiye. Our overall prevalence was higher than that reported in other recent national studies and was driven predominantly by NNRTI-associated mutations among treatment-naïve individuals. The complete absence of INSTI resistance reaffirms the robust efficacy of contemporary INSTI-based first-line regimens in this setting. Furthermore, the exceptionally high prevalence of the S68G accessory mutation, consistent with recent national data, suggests a distinctive virological pattern within the Turkish HIV-1 population whose epidemiological origins warrant further phylogenetic investigation. These findings support the value of baseline genotypic resistance testing prior to ART initiation, particularly when rilpivirine-based or long-acting regimens are being considered. Continued genotypic surveillance in the Aegean region, ideally incorporating next-generation sequencing and phylogenetic analysis, would help clarify the clinical significance of the observed resistance patterns and inform regimen selection as the Turkish epidemic continues to diversify.

## Data Availability

The datasets generated and analyzed during the current study are not publicly available due to the inclusion of sensitive patient information that cannot be fully de-identified. Requests for access to the anonymized dataset may be directed to the corresponding author on reasonable request.
